# Acoustic signal analysis of instrument–tissue interaction for minimally invasive interventions

**DOI:** 10.1007/s11548-020-02146-7

**Published:** 2020-04-22

**Authors:** Daniel Ostler, Matthias Seibold, Jonas Fuchtmann, Nicole Samm, Hubertus Feussner, Dirk Wilhelm, Nassir Navab

**Affiliations:** 1 Minimally Invasive Interdisciplinary Therapeutical Intervention, Technical University Munich, Munich, Germany; 2 Chair for Computer Aided Medical Procedures and Augmented Reality, Technical University Munich, Munich, Germany; 3 Research in Orthopedic Computer Science Group, Balgrist University Hospital, University of Zurich, Zurich, Switzerland; 4 Department of Surgery, Klinikum rechts der Isar, Technical University Munich, Munich, Germany

**Keywords:** Minimally invasive surgery, Visceral surgery, Deep learning, Audio analysis, Spectrogram, Audio perception

## Abstract

****Purpose**:**

Minimally invasive surgery (MIS) has become the standard for many surgical procedures as it minimizes trauma, reduces infection rates and shortens hospitalization. However, the manipulation of objects in the surgical workspace can be difficult due to the unintuitive handling of instruments and limited range of motion. Apart from the advantages of robot-assisted systems such as augmented view or improved dexterity, both robotic and MIS techniques introduce drawbacks such as limited haptic perception and their major reliance on visual perception.

****Methods**:**

In order to address the above-mentioned limitations, a perception study was conducted to investigate whether the transmission of intra-abdominal acoustic signals can potentially improve the perception during MIS. To investigate whether these acoustic signals can be used as a basis for further automated analysis, a large audio data set capturing the application of electrosurgery on different types of porcine tissue was acquired. A sliding window technique was applied to compute log-mel-spectrograms, which were fed to a pre-trained convolutional neural network for feature extraction. A fully connected layer was trained on the intermediate feature representation to classify instrument–tissue interaction.

****Results**:**

The perception study revealed that acoustic feedback has potential to improve the perception during MIS and to serve as a basis for further automated analysis. The proposed classification pipeline yielded excellent performance for four types of instrument–tissue interaction (muscle, fascia, liver and fatty tissue) and achieved top-1 accuracies of up to 89.9%. Moreover, our model is able to distinguish electrosurgical operation modes with an overall classification accuracy of 86.40%.

****Conclusion**:**

Our proof-of-principle indicates great application potential for guidance systems in MIS, such as controlled tissue resection. Supported by a pilot perception study with surgeons, we believe that utilizing audio signals as an additional information channel has great potential to improve the surgical performance and to partly compensate the loss of haptic feedback.

## Introduction and related work

In the past decades, minimally invasive surgery has become a standard technique in visceral interventions. Procedures such as cholecystectomy, appendectomy or adrenalectomy are nowadays most commonly carried out in a laparoscopic technique. By accessing the operation area only through small incisions, traumata can be minimized, infection rates reduced, and hospitalization times shortened [29].

Although nowadays systems such as 3D high-resolution laparoscopes improve spatial perception and visualization during MIS, it is not proven that these system reduce complication rates [10]. Since surgeons rely mainly on visual- and partly haptic perception, we postulate that surgical performance could be improved by adding auditory feedback during laparoscopic interventions. More precisely, we believe that a system which is able to guide the surgeon during tissue resection by analyzing the acoustic emissions generated by instrument–tissue interaction during diathermy can improve the safety and precision of interventions. In the following paragraphs, we briefly summarize the state of the art of acoustic signal analysis for medical use-cases followed by the preprocessing and machine learning methods utilized for audio-based classification. This work does not claim to present an operational solution ready to be deployed in the surgical OR. Rather it is intended to present a novel concept which combines audio signal processing and minimally invasive surgery.

### Acoustic emission analysis for medical applications

Acoustic signals are always present in the operating theater, e.g., acoustic signals generated by the surgeon’s interaction with the patient, such as diathermy sounds, continuous signals from surgical devices such as heart monitors or alarms and notification sounds. By recording and analyzing acoustic signals in a diagnostic or interventional environment, highly dense information about the current state and events can be captured using a low-cost sensor interface.

One example for a diagnostic use-case of acoustic signal analysis originates from chest medicine, using different diagnosis techniques based on stethoscope signals. Shkelev et al. [30] proposed a system for the automated analysis of cardiosignals by recording the heart sounds with an electret microphone. They used temporal and spectral methods to analyze the state of the cardiovascular system under normal conditions and increased loads. The system developed by Marshall et al. [17] uses signal processing algorithms to enable non-specialists to screen for pulmonary fibrosis. Furthermore, algorithms were developed to compute vital body function measures such as pulmonary arterial pressure from recorded heart sounds with high accuracies [34].

Also in orthopedics, acoustic signals have been used for diagnosis and guidance. Rangayyan et al. introduced a technique called Vibroarthography (VAG) which is characterized by recording acoustic emissions from knee joints in order to detect malicious joint conditions. They demonstrated that various degrees of chondromalacia and meniscal lesions can be detected by performing a frequency analysis on the audio signal recorded with surface microphones from the patient’s skin [26]. Machine learning approaches have been introduced to classify VAG signals with high accuracy rates [1, 11, 20].

Illanes et al. proposed a novel method to characterize medical interventional devices insertion events by attaching an acoustic sensor to the proximal part of the apparatus [8]. They showed that the method allows to identify transitions between different types of tissues during needle insertion. This concept was applied in further research in an experimental setup to analyze the influence of different insertion depths and the interaction of the surrounding soft tissue with the needle surface to the resulting measurements [16]. Moreover, the tissue-layer crossing identification capabilities of the system were successfully tested with the application of Veress needle placement for minimally invasive interventions [28].

### Machine learning for audio classification

Advances in the research field of Automatic Speech Recognition (ASR), which has gained a lot of interest in recent years, were also beneficial for the emerging field of sound event classification. Machine learning algorithms were successfully applied to detect sound events in everyday life, such as urban sounds [23] or musical genres [21].

Recently, methods used for sound classification have shifted from traditional approaches such as Gaussian Mixture Models (GMMs) or Hidden Markov Models (HMMs) with handcrafted features or Mel Frequency Cepstrum Coefficients (MFCC) [6, 22] to deep learning methods, such as CNNs, and deep recurrent neural networks (RNNs). As these new techniques outperformed the state-of-the-art models in speech and language processing, deep learning methods were also applied to acoustic scene recognition challenges. Li et al. [13] and Dai et al. [3] tested various feature sets including MFCCs with different deep learning algorithms such as deep neural networks and deep RNNs.

Their results showed that with large feature sets, deep learning methods outperform traditional classification methods and achieve best performances in comparison with conventional algorithms. Cakir et al. used frame-based spectral features to train a deep neural network classifier for environmental sound detection [2] which improved the classification accuracy compared to a baseline HMM classifier by 19%.

**Fig. 1:**
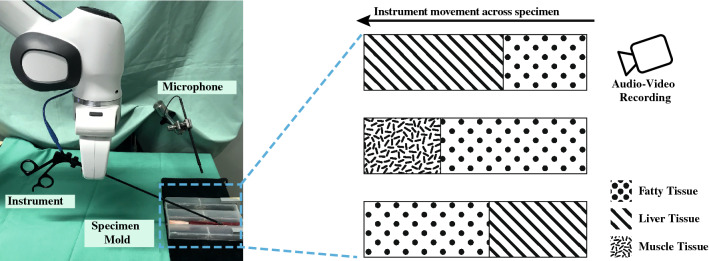
Experimental setup with a *Franka Emika Panda* robot and the attached electrode, a microphone as well as multiple tissue types aligned to specimens within the specimen mold. The instrument, wired to a electrosurgical unit, is dragged along the surface of each specimen with constant speed. Additional audio and video for visual ground-truth determination was recorded using a separate camera

CNNs together with spectrogram features were first applied by Zhang et al. [37] to the task of sound event recognition. The spectrogram-based approach has the advantage of naturally capturing the sound information in a two-dimensional feature space. In contrast to conventional frame-based one-dimensional features, more information than just a slice of spectral information can be captured [5]. While spectrogram-based features retain more information about the original audio source compared to most hand-crafted features, they are of lower dimension than raw audio which is usually sampled with a rate of over 40 kHz [36]. Therefore, the approach is a good compromise between dimensionality reduction and information preservation and is nowadays widely adopted for audio classification tasks.

There are different types of auditory images used in audio classification. Mel-spectrogram-based approaches have been successfully applied to the task of musical genre and mood recognition [14]. Constant-Q transform-based spectrograms have been used to classify urban sounds [15]. Valada et al. [35] implemented a Short-Time Fourier Transform (STFT) spectrogram-based approach for robotic terrain classification based on the interaction of the robot’s wheels with the underground. They compared different window lengths for spectrogram generation and achieved classification accuracies of up to 99%. Pons et al. showed that even with randomly distributed weights, a CNN architecture is able to extract meaningful features from an auditory image [24].

The following section presents the methodology of this work and is separated into two subsections, a perception study and a proof-of-concept system for instrument–tissue interaction classification.

## Methodology

The aim of the first experiment was to investigate whether acoustic signals from the abdomen inside can, when transmitted, improve the perception of the intervention. Therefore, we conducted a user study by asking surgeons to identify the transition between different types of issue by listening to audio recordings of a standardized acquisition setup explained in “Perception study” section. Furthermore, all participants were presented with a questionnaire consisting of 13 questions about the subjective perception of audio signals in minimally invasive interventions.

The objective of the second experiment is to investigate whether the signals, when recorded, can be used as basis for a learning-based automated classification system which can further support the surgeon during the intervention. This twofold approach can be seen as an analogy to the visual examination of conventional medical imaging, e.g., radiographs, by a human observer and the automatic detection of lesions by a learning-based system.

### Perception study

Within its audible scope, the auditory system of human beings is particularly sensitive regarding relative changes in signals (e.g., changes in timbre, pitch, loudness) [27]. Hence, the first experiment focuses on the contextual perception of sounds caused by minimally invasive electrosurgical procedures. Therefore, 27 specimens were prepared, each consisting of two different tissue-type combinations with various lengths aligned successively in a mold yielding to different transition points. The porcine specimens included liver, muscle and fatty tissue. The complete setup is shown in Fig. 1.

**Fig. 2:**
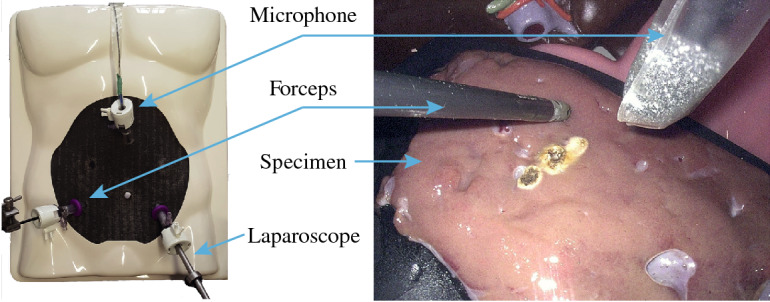
External view (left) and internal view through laparoscope (right) of the experimental setup using a box trainer. Microphone and sterile cover, laparoscope and laparoscopic forceps for the coagulation of specimens are inserted via trocars

While applying only little pressure, a monopolar biopsy forceps was dragged along the surfaces of the specimens at constant speed, coagulating the tissues with the highest power setting available on the electrosurgical unit. A *DPA d:screet 4060 Heavy Duty* miniature condenser microphone was positioned in the middle above the mold for the acquisition of the audio signal with uncompressed high quality and a sampling rate of 44.1 kHz using the audio stream input/output (ASIO) driver protocol. Subsequently, the audio recording was manually synchronized with audio and video recordings of an additional camera on a frame-based level (24 fps). The videos served as a visual ground truth in order to individually determine the transition point within each specimen. The transition point, i.e., the ground truth, was noted as point in time within each recording.

With an ASIO sound driver latency of approximately 16 ms for a buffer sample size of 512, generated audio recordings were presented directly and without any preprocessing to multiple surgeons who were asked to identify the transition point between the two tissues types within each specimen solely by listening to the audio signal through headphones. Finally, transition points identified by the surgeons were compared to the ground truth based on the captured camera feed and differences were evaluated.

Subsequently, the participating surgeons were given a questionnaire to answer 13 questions about acoustic perception in minimally invasive interventions and identifying instrument–tissue interaction in acoustic signals.

**Fig. 3:**
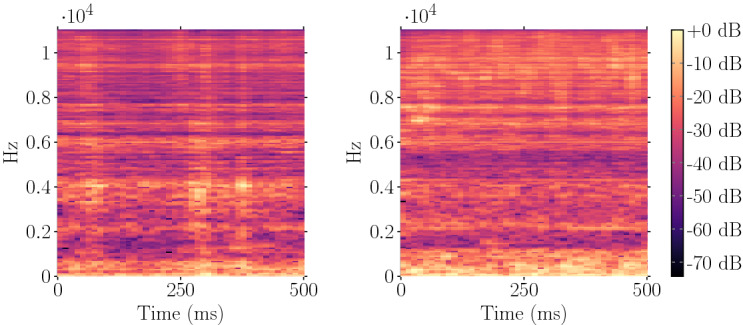
Two example spectrogram representations of the classes *fascia* (left) and *fat* (right); the *x*-axis represents time in milliseconds, the *y*-axis the Mel-frequency scales, the pixel intensity the amplitude in decibel (dB)

### Acoustic-based instrument–tissue interaction classification

In the following sections, we present a proof-of-concept approach for a classification system which was implemented to automatically detect and classify instrument–tissue interaction. We present the experimental setup for data acquisition and introduce a preprocessing and transfer-learning-based classification pipeline. Deep learning-based audio classification methods, presented in “Machine learning for audio classification” section, have been reported to achieve great results in the field of environmental sound classification, especially under noisy conditions [35]. In the following sections, we present an approach to transfer the techniques to the medical use-case of instrument–tissue interaction classification.

#### Data acquisition

For data acquisition, a similar setup as described in “Perception study” section was used to record the acoustic signals. To simulate MIS, specimens of porcine tissue were placed inside a surgical box trainer. With its diameter of 5.4 mm, the miniature microphone is small enough to be inserted into a surgical trocar which is necessary for recording audio signals from the inside of a cavity, such as the inflated human abdomen during visceral MIS.

For a future-perspective application in surgical procedures, sterility of all the utilized devices in contact with the patient has to be considered. Despite its rugged construction, the used microphone is not autoclavable. Therefore, several sterile covers have been tested and evaluated with respect to their acoustic transmission characteristics. A laparoscopic ultrasound cover was chosen as it best preserves high-frequency content in the recorded audio signal. To avoid scratch and friction noise, before applying the cover, a foam wind cover was placed over the microphone capsule. With its sleeve-like shape, it fits the form of the microphone nicely and enables easy intra-abdominal insertion through the trocar. In order to replicate the different basic tissues present in the abdominal cavity, specimens of fascia and fatty tissue—representative for connective tissue, liver tissue, as well as muscular tissue were chosen. Figure 2 shows the experimental setup and illustrates the placement of microphone with sterile cover, laparoscope, and forceps.

A standard laparoscopic biopsy forceps was connected to the electrosurgery unit and used to apply current to the tissue probe. To cover the operation range of the electrosurgical device, three different power settings (low, mid-range and high) were applied for both cutting and coagulation mode. The sound clips were recorded with an average length of about 2 s, which was chosen in accordance with the average application length of electrosurgery found by Meeuwsen et al. [19]. The final data set consists of 1758 individual sound clips.

#### Signal processing

We chose a spectrogram feature-based approach, depicted in Fig. 3, as auditory image features have been shown to yield superior classification performances [25]. A rectangular sliding window function was applied to the individual sound clips to compute log-mel-spectrograms from the data set. The window length was thereby varied between 300, 500 and 1000 ms with an overlap of 75% which resulted in a total number of 60.880, 34.052, and 13.970 samples, respectively.

Spectrograms are two-dimensional visualizations of spectral sequences with time on the abscissa and frequency on the ordinate. The color intensity of each pixel refers to the amplitude of the respective frequency. In the first step, Short-Time Fourier Transformation (STFT) was computed for each windowed segment of the audio clip by applying:

1 $$\begin{aligned}&X(i,j) = \sum _{p = 0}^{N_f-1}x[n] w[n - j] \exp \left( -p \frac{2\pi k}{N_f}n\right) , \quad \nonumber \\&\quad p = 0,\ldots , N_f-1 \end{aligned}$$

where *x*[*n*] denotes the signal consisting of $N_{f}$ samples, $w[n - j]$ the windowing function at frame $n-j$ explained in Eq. 2, *p* is the iteration variable, and 2$\pi k$ is the frequency. The result *X* is a matrix containing the magnitude of frequency bin *i* at frame *j*. We used a window length of $N_{f} = 2048$ samples for STFT computation. The step size of the sliding window was set to 512 samples which results in a window overlap of 75%. To compensate for the Gibbs effect, a *Hann* windowing function was applied:

2 $$\begin{aligned} w[n] = \frac{1}{2} \left[ 1- \cos \left( 2 \pi \frac{n}{M - 1}\right) \right] , \quad n = 0,\ldots ,M-1 \end{aligned}$$

Furthermore, the matrix was converted from energy to power spectrogram by squaring the amplitude. Additionally, the power spectrogram was mapped to a decibel scale by computing:

3 $$\begin{aligned} X_{\mathrm{pow}}(i,j) = 10 \, \log _{10}(X(i,j)^{2}) \end{aligned}$$

The signal was filtered in the spectral domain with a triangular-shaped Mel filter bank. These filters provide an approximation to the nonlinearities of the human cochlea and are also the basis for the computation of MFCCs. The applied filters are spaced evenly on the Mel scale introduced by Stevens et al. [31] which can be calculated from frequency by:

4 $$\begin{aligned} f_{\mathrm{mel}} = 2595 \, \log _{10}\left( 1 + \frac{f}{700}\right) \end{aligned}$$

The Mel filter bank can be seen as a simplified version of the gammatone filter bank which has been shown to be highly correlated with natural sound signals. Its application produces a sparse, high-resolution spectrogram from the audio source [12]. A total number of 256 Mel filter bands were used to combine the Fast Fourier Transform (FFT) bins into Mel-frequency bins. We computed spectrograms for a frequency range from 0 to 11,025 Hz. The spectrograms were normalized by $X_{\mathrm{norm}, \mathrm{mel}} = {(X_{\mathrm{mel}} - \mu )} / {\sigma }$, where ($\mu$) is mean and ($\sigma$) is the standard deviation computed over the entire data set.

Figure 3 illustrates example log-mel-spectrograms computed from clips of the classes *fascia* and *fat* with a window length of 500 ms.

**Table 1:** Overall test accuracy for different spectrogram configurations

	$\Delta t = 300$ ms (%)	$\Delta t = 500$ ms (%)	$\Delta t = 1000$ ms (%)
$f_{\min } = 2$ kHz	86.25	88.88	89.90
$f_{\min } = 0$ kHz	84.62	88.17	89.56

#### Network architecture and training

For the proof-of-concept system, we applied a transfer learning approach which has been shown to work effectively for CNN architectures [33]. We extracted log-mel-spectrograms with dimensions $299 \times 299 \times 3$ from the entire data set and split the data into training, validation and test set with a distribution of 80%, 10% and 10%, respectively. We chose the deep convolutional neural network architecture Inception-v3 [32] which has shown to yield excellent performance on log-mel-spectrogram-based audio classification [7]. The network was pre-trained on 14,197,122 images and 1000 classes of ImageNet [4], and was used to extract a descriptive feature vector from the intermediate spectrogram representation. We used a mini-batch size of 32 according to Masters et al. [18] to train a single fully connected layer with five output classes on the CNN features with dimensions $1 \times 2048$. We applied a RMSprop optimizer with fixed learning rate for minimizing a softmax cross-entropy cost function *H*(*y*, *p*):

5 $$\begin{aligned} H(y,p) = -\sum _{c = 1}^{M}y_{o,c} \log (p_{o,c}) \end{aligned}$$

where *M* denotes the total number of classes, *y* is a binary indicator if class label *c* is the correct prediction for observation *o* and *p* is the predicted probability that observation *o* is of class *c*. We implemented early stopping regularization to avoid overfitting of the training routine.

## Results and evaluation

### Evaluation of the perception study

For better comparability, all audio recordings were cut to a length of 12 s per capture, i.e., representing the coagulation sounds across the tissue samples. To learn about the potentials of acoustic signals in MIS and to investigate whether different types of tissues can be discriminated, 6 surgeons, trained in the field of MIS, estimated the transition points between the two tissue types within each recording solely by listening. In total average, they missed the reference marker, prior defined by visual annotation, by 1079 ms. However, the median of all 162 single measurements amounts only to 472 ms, indicating a rather precise distinguishability.

The evaluation of the questionnaire revealed that the surgeons rate the acoustic feedback in MIS significantly worse than in traditional open surgery. They reported that acoustic feedback has the potential to improve the perception of MIS. Showing that surgeons are able to extract useful information from acoustic signals recorded from the operation area supports the hypothesis that these signals can be used for further automated analysis to support surgeons during challenging interventions.

### Evaluation of the classification pipeline

In the following paragraphs, we evaluate the performance of the classifier applied to the problem of acoustic-based instrument–tissue interaction classification. We compare different spectrogram configurations and analyze the discriminability of tissue types and electrosurgery operation modes.

#### Comparison of spectrogram configurations

To analyze the performance of our model under different preprocessing settings, we varied the length of the rectangular sliding window. A detailed frequency analysis of the raw audio signal showed that low-frequency content mostly contained environmental noise. Therefore, we additionally compared the performance of the network with full frequency scale of 0–11 kHz and reduced frequency scale of 2–11 kHz. Table 1 shows the results of our evaluation with the rows corresponding to varying frequency scales and columns to different sliding window lengths.

The trained model achieves accuracies up to 89.90% on the test set. With larger window length the accuracy improves, but execution time of one classification step for the deployed model increases, respectively. Therefore, a reasonable window length has to be chosen as a trade-off between execution time and model performance. For further analysis, we defined a window length of 500 ms and frequency scale 2–11 kHz to balance execution time and classification accuracy.

#### Discriminability of tissue types

**Fig. 4:**
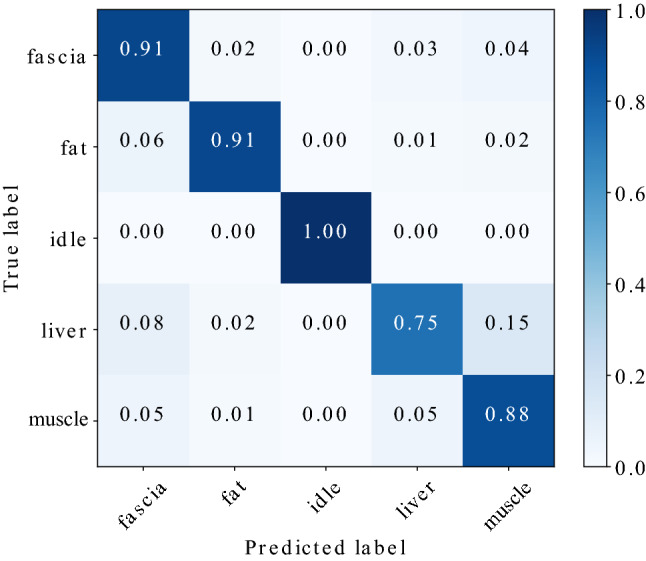
Confusion matrix of spectrogram configuration [${\text {windowlength}} {=} 500$ ms, $f_{\min }{=} 2000$ Hz] for the test set

**Fig. 5:**
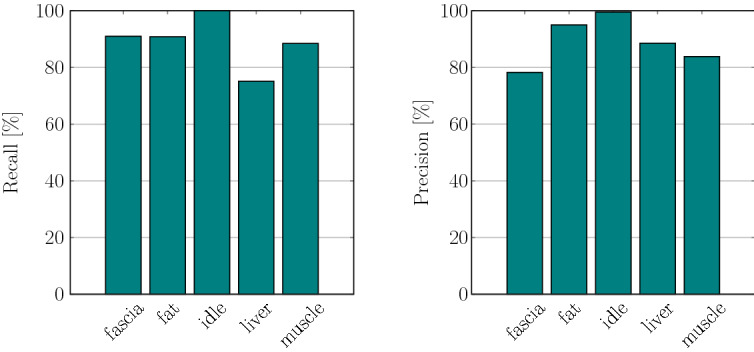
Per-class recall and precision of the network on a spectrogram configuration with window length of 500 ms and reduced frequency range

The classification results in terms of discriminability of tissue types are condensed as confusion matrix in Fig. 4. The network is able to distinguish between ‘idle’ state and application of diathermy taking place with a high true-positive rate of 100%. The class ‘fat’ also reaches fairly high values of 91%. Furthermore, it can be observed that the model confuses the classes ‘liver’ and ‘muscle’ with confusion probabilities of 15% and 5%, respectively, for this spectrogram configuration. The lowest true-positive rate was obtained for the ‘liver’ class.

Figure 5 compares per-class recall and precision for the test data set. The network achieved an average recall of 89.10% and an average precision of 89.04%. The F1-score reaches a value of 89.07%, accuracy was measured as 88.88%.

**Fig. 6:**
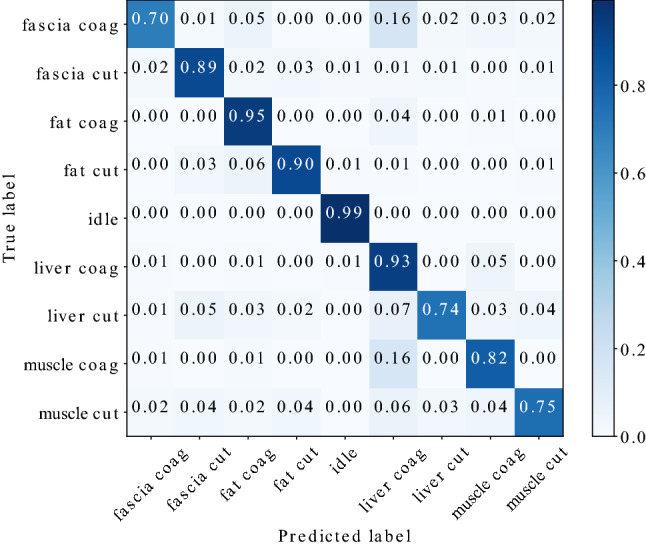
Confusion matrix of spectrogram configuration [${\text {windowlength}} = 500$ ms, $f_{\min } = 2000$ Hz] with 9 classes representing different tissue types and electrosurgical operation modes

#### Discriminability of electrosurgery operation modes

Additionally, the model’s capability to distinguish between cutting and coagulation mode for each class was evaluated, which is illustrated in Fig. 6. During data acquisition, audio recordings of both electrosurgical operation modes have been acquired with equal distribution. Therefore, no bias is introduced by splitting the data set into 9 classes.

Taking the electrosurgery mode into account, the overall accuracy reaches a value of 86.40%. Average precision equals to 86.75%, average recall to 85.27%, and F1-score to 86.01%. The confusion matrix reveals that the network performs well on separating the operation modes. For example, the network confuses the classes ‘liver’ and ‘muscle’ more likely than cutting and coagulation.

**Fig. 7:**
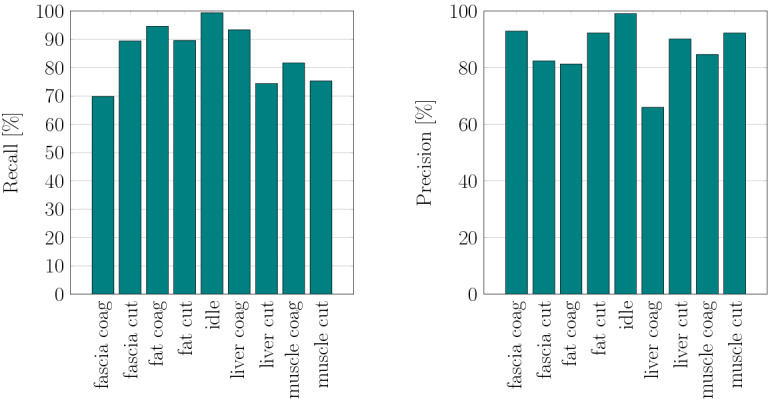
Per-class recall and precision of the network on a spectrogram configuration with window length of 500 ms and reduced frequency range for a data set configuration with 9 classes

Figure 7 illustrates per-class recall and precision for the model trained on 9 classes.

## Discussion

The perception study suggests that tissue-related acoustic differences during diathermy are detectable merely by listening. Even though the estimated time stamps deviate slightly from the references and individual reaction time has to be considered as an additional delay of less than 250 ms on average, surgeons could derive additional information through audio signals which are currently not provided during MIS [9]. The evaluation of the questionnaires revealed that acoustic signals recorded in MIS have the potential to both improve the perception of the intervention and serve as the basis for further supportive automated analysis. Because coagulation sounds are audible for traditional open surgery and supported by the perception study, we believe that the transmission of sound from inside the abdomen is not irritating but rather a useful augmentation in a minimally invasive surgical scenario.

The results of the classification framework look promising in terms of discriminability of tissue and operation modes. However, to transfer our experimental methodology to surgery, certain shortcomings of our experimental setup have to be addressed. Inter alia the acoustic properties of the used box trainer are different to a CO_2_-insufflated human abdomen in respect to insulation, shape, volume, material, reflective areas and potential sound sources. An in-depth analysis of the differences can only be achieved through in-vivo experiments. Such in-vivo animal studies are one of our major next steps, while this present work will form the scientific basis for a ethical approval application of animal experiments.

Further research is required to determine which physical tissue properties influence the sound generation during diathermy.

As we had to limit our experimental setup to a box trainer for now, the weak acoustic insulation of the latter brings a few drawbacks to the experiment.

Regulations demand activation and alarm tones for electrosurgical generators which cannot be turned down arbitrarily and are slightly audible in the recordings from the experimental setup (“Data acquisition” section). We analyzed the recordings and found the characteristic alarm tones to be sine waves with 2020 Hz and 3035 Hz for cutting and 1380 Hz and 2270 Hz for coagulation mode. We added an additional preprocessing step to filter out the beeps with IIR-based notch filters but could not observe increasing classification performance.

Since also environmental noise is audible on the recordings, we applied a hard low-cut at 2 kHz and used only the signal above this frequency threshold. The spatial resolution in the spectrogram representation is hence increased for the remaining 163 Mel-bins. The filtering of the background noise and the improved bin-to-pixel-ratio increased the classification accuracy for sliding window lengths above $\Delta t> {300}$ ms (see Table 1) and was hence applied prior all analyses.

The model performance could possibly be improved by applying augmentation strategies, such as time stretching, tempo or pitch modulation or adding noise and reverberation to the audio data to simulate different environments. Moreover, different model architectures have to be evaluated to further improve the performance of the classification algorithm.

## Conclusion

In this paper, we present a novel approach for intra-abdominal acoustic analysis in minimal invasive surgery. In a user study, we investigated whether the transmission of audio signals from inside of the abdomen during MIS has potential to improve the perception during procedures and observed that the participating surgeons are able to intuitively derive additional information from the signals. We furthermore introduced a first concept which uses the acquired signals for the classification of instrument–tissue interaction during diathermy by training a log-mel-spectrogram-based CNN classification pipeline on acoustic signals recorded directly from the operation area. Our model reached accuracies of up to 89.90% on a data set acquired in an experimental setup. In addition, we evaluated the network’s capability to distinguish between electrosurgical operation modes which resulted in an overall classification accuracy of 86.40%.

Results of the proposed system indicate potentials inter alia for the use as a guidance system or to enable tissue-related energy settings and security circuits; all reducing patients risk while improving the outcome.
